# Biocompatible, Electroconductive, and Highly Stretchable Hybrid Silicone Composites Based on Few-Layer Graphene and CNTs

**DOI:** 10.3390/nano11051143

**Published:** 2021-04-28

**Authors:** Marie N. Barshutina, Valentyn S. Volkov, Aleksey V. Arsenin, Dmitriy I. Yakubovsky, Alexander V. Melezhik, Alexander N. Blokhin, Alexey G. Tkachev, Alexander V. Lopachev, Vladislav A. Kondrashov

**Affiliations:** 1 Center for Photonics and 2D Materials, Moscow Institute of Physics and Technology, 141700 Dolgoprudny, Russia; volkov.vs@mipt.ru (V.S.V.); arsenin.av@mipt.ru (A.V.A.); dmitrii.yakubovskii@phystech.edu (D.I.Y.); 2 Institute of Translational Biomedicine, Saint-Petersburg State University, 199034 Saint-Petersburg, Russia; 3 Department of Technology and Methods of Nanoproducts Manufacturing, Tambov State Technical University, 392000 Tambov, Russia; nanocarbon@rambler.ru (A.V.M.); cha-cha@rambler.ru (A.N.B.); postmaster@kma.tstu.ru (A.G.T.); 4 Laboratory of Clinical and Experimental Neurochemistry, Research Center of Neurology, 125367 Moscow, Russia; lopachev@neurology.ru; 5 Skolkovo Institute of Science and Technology, 121205 Moscow, Russia; v.kondrashov@skoltech.ru

**Keywords:** graphene, carbon nanotubes, polydimethylsiloxane, hybrid nanocomposites, bioelectronics

## Abstract

In this paper, we report a cost-effective and scalable approach to produce highly homogeneous graphene and CNT-based silicone composites with potential applications in diverse fields of research, including biosensors and wearable electronics. This approach includes the fabrication of hybrid fillers based on few-layer graphene and CNTs by water solution blending and manufacturing of graphene/CNT/PDMS composites through calendering in a three-roll mill. The influence of processing parameters, the graphene/CNT ratio, and hybrid filler loading was thoroughly investigated, and the optimal parameters for producing hybrid composites with superior electrical and mechanical properties were found. It was also confirmed that the graphene/CNT hybrid system exhibits a synergistic effect of non-covalent interactions between graphene sheets and CNT sidewalls. This synergistic effect prevents the aggregation of graphene sheets, facilitates the dispersion of graphene and CNTs in the silicone matrix, and contributes to the superior properties of hybrid composites compared to composites with either of these fillers alone.

## 1. Introduction

In recent decades, a great deal of attention has been paid to the development of biomaterials for application in various fields, such as health monitoring, clinical surgery, tissue engineering, and drug delivery [1,2,3]. Most commonly used biomaterials are based on polymers due to their organic and versatile nature. In order to obtain polymer biomaterials with certain advantageous properties, two approaches are usually employed [4]: (1) the alteration of the chemical constitution and (2) the incorporation of a second phase (filler) in the polymer matrix. The second approach allows altering the initial properties of polymers in a fairly wide range so that polymer composites with unique electrical, mechanical, optical, magnetic, and biological properties can be obtained.

The stretchable and conductive polymer composites are of particular interest for use in bioelectronics, especially for the manufacturing of biosensors, and bioimplants [5]. The sufficient levels of conductivity and stretchability of these composites are achieved by incorporating the conductive fillers into the matrix of elastic polymers, such as silicones, natural rubber, hydrogels, and polypeptides [6]. As for the conductive filler, two categories of fillers are commonly used: metals and carbons (including graphene (G), carbon nanotubes (CNTs), carbon fibers (CF), and carbon black (CB)) [7,8].

During the last decade, graphene and CNT-based elastomeric composites have attracted much attention of researchers and have been considered as the most promising for biomedical applications [9,10]. A unique structural feature of these composites is their immense interfacial area between matrix and filler, which causes strong molecular interactions between two constituents, and contributes to the improvement of many macro-level composite properties. In particular, compared to conventional bioelectrode materials, CNT and graphene-based composites have increased flexibility, tensile strength, specific capacitance, signal-to-noise ratio (SNR), electrical conductivity, thermal stability, etc. [11,12]. However, to achieve superior properties of composites, proper dispersion and good interfacial bonds between filler nanoparticles and the polymer matrix have to be guaranteed, which is quite a challenging task due to the tendency of nanoparticles to form agglomerates because of strong van der Waals interactions.

Various methods have been proposed to enhance the dispersion of graphene or CNTs in a polymer matrix, including ultrasonic treatment, stirring, ball milling, shear mixing, solution blending, use of surfactants, and chemical functionalization [13,14]. Nevertheless, there remains much room for improvement, especially when it comes to the fabrication of composites for biomedical applications.

Recently, three-dimensional G/CNT hybrid materials have been proposed for the manufacturing of highly homogeneous composites with potential applications in diverse fields of research, including supercapacitors, batteries, and biosensors [15]. The most important feature of these hybrid materials is a non-covalent π-π stacking interaction operating between sp^2^-hybridized graphene sheets and CNT sidewalls, which prevent the aggregation of individual graphene sheets and promote their dispersion in polymer composites.

Although many researchers have studied the polydimethylsiloxane (PDMS) composites with either CNTs or graphene fillers, there have been few studies on properties of silicone composites containing CNT–graphene hybrid materials [16,17,18,19,20,21,22,23,24,25,26]. In particular, Hu et al. [16] investigated the dispersion of G/CNTs hybrid fillers in silicone rubber. They reported that CNTs and graphene together form a well-dispersed conductive network in the silicone matrix; furthermore, a strong synergistic effect of these two fillers on electrical conductivity and thermal diffusivity was observed. Pradhan et al. [17] also reported on the synergistic effect between graphene and CNTs in silicone matrix. The authors found that the G/CNT hybrid composites exhibited improved mechanical, thermal, and chemical properties compared to single-filler composites. Particularly, a significant enhancement in tensile strength, Young’s modulus, thermal stability, and solvent resistance has been shown. Another group of researchers (Oh et al. [18]) studied the hybrid silicone composites with small fractions of CNTs and thermally reduced graphene (TRG) for potential applications in flexible and stretchable electronics. According to the results of their research, the interconnections between CNTs and TRG in the silicone matrix play a synergistic role in increasing the electrical conductivity and lowering the electrical percolation threshold. Recently, Yang et al. [20] conducted studies on the static and dynamic strain sensing behaviors of G/CNT/PDMS composites. The obtained results showed that the resistance response of these composites under continuous cyclic loading exhibited good stability, recoverability, and repeatability, which indicates a high potential for dynamic monitoring applications.

Overall, the discussed studies have demonstrated that the synergistic effect of CNT and graphene interactions facilitates their dispersion in the silicone matrix and contributes to superior properties of hybrid composites compared to composites with either of these fillers alone. However, the composite fabrication techniques employed in these studies have some significant drawbacks for biomedical applications. In particular, the production of G/CNT hybrid fillers has been carried out mainly through a solution blending method involving the use of solvents (tetrahydrofuran [16,17,18,19], hexane [20,21], acetate [23], toluene [24], etc.), which can affect the toxicity of ultimate composites and contribute to the deterioration of their electrical and mechanical properties. Moreover, solution blending is a multi-stage and small-scale process that is mainly used in laboratories for producing polymer composites but barely adaptable to industry.

Thus, the development of a simple, cost-effective, and scalable technique for the production of G/CNT hybrid silicone composites is still a key challenge. In this paper, we proposed a new approach to address this challenge, which involves the fabrication of G/CNT hybrid fillers by water solution blending and manufacturing of G/CNT/PDMS composites by calendering in a three-roll mill. The proposed technique does not involve the use of any toxic solvents and offers several considerable advantages, such as low cost, superior uniformity of product, enhanced production speeds, and feasibility of constant in-process monitoring.

In the framework of this paper, we also investigated the influence of the processing parameters and components ratio upon the mechanical, electrical, and morphological properties of silicone composites based on G/CNT hybrid fillers. The experimental results are compared to those of composites based on either CNTs or graphene alone, and a synergistic effect of G/CNT hybrid fillers on properties of silicone composites is discussed.

## 2. Experimental Section

### 2.1. Materials

Purified multi-walled carbon nanotubes (Taunit M; purity: ≥99%, OD: 10–30 nm, ID: 5–15 nm, length: ≥2 µm) and water suspension of few-layer graphene (FLG) (number of layers: 3–5, particle size: 2–10 µm) were supplied by NanoTechCenter, Ltd., Tambov, Russia. The Sylgard 184 silicone elastomer kit (10:1 mix ratio) was supplied by Dow Corning, Midland, MI, USA.

A human neuroblastoma SH-SY5Y cell culture for cytotoxicity research was supplied by ATCC^®^, Manassas, VA, USA. Eagle’s Minimum Essential Medium (EMEM) and 1% penicillin-streptomycin solution were purchased from PanEco, Moscow, Russia. 10% fetal bovine serum (FBS) was supplied by BioSera, Ringmer, UK. 1 µM retinoic acid was purchased from Sigma, St. Louis, MO, USA. 3-(4,5-dimetylthiazol-2-yl)-2,5-diphenyl tetrazolium bromide (MTT) reagent was supplied by Dia-M, Moscow, Russia. Dimethyl sulfoxide (DMSO) was supplied by Applichem, Omaha, NE, USA.

### 2.2. Preparation

The water suspension of FLG was divided into six parts, and five of them were filled with a proper amount of CNTs to produce G/CNT (9:1, 8:2, 6:4, 4:6, and 2:8 mass ratios) hybrids. All mixtures were intensively stirred in an ultrasonic bath (50 W, 80 kHz) for 20 min, and then all samples (including a pristine graphene sample) were dried in a vacuum oven at 110 °C until constant weight was achieved. Finally, the dried samples were processed in a disintegrator for 4 min to obtain a fine, homogeneous and non-agglomerated product.

Further, the upper concentration limits of graphene, CNTs, and G/CNT hybrid fillers in silicone composites were studied. The upper concentration limit is the maximum amount of filler that can be incorporated in the silicone matrix while maintaining a visible uniformity of filler distribution. Apparently, these values directly depend on the bulk density (*ρ_b_*) of fillers. The upper concentration limit of 10 wt.% was reached for low-density fillers such as CNTs (*ρ_b_* = 0.025 g/cm^3^) and hybrids with G/CNT mass ratios of 2:8 (*ρ_b_* = 0.086 g/cm^3^) and 4:6 (*ρ_b_* = 0.131 g/cm^3^). The upper concentration limit of 20 wt.% was reached for high-density fillers such as graphene (*ρ_b_* = 0.29 g/cm^3^) and hybrid with the G/CNT mass ratio of 9:1 (*ρ_b_* = 0.184 g/cm^3^). For the middle-density fillers such as hybris with G/CNT mass ratios of 6:4 (*ρ_b_* = 0.150 g/cm^3^) and 8:2 (*ρ_b_* = 0.168 g/cm^3^), the upper concentration limit was found to be 15 wt.%.

A three-roll mill (TRM) (Exact 120 S, EXACT GmbH, Remscheid, Germany) was then used for fabrication of silicone composites based on graphene, CNT, or hybrid fillers with various G/CNT mass ratios and concentrations. In order to optimize the TRM processing parameters, several mixing trials were carried out using composite samples with 10% wt. of G/CNT (8:2) hybrid filler. Each trial was performed in either 3 or 6 steps and with varied gaps (5, 10, 20, 40, 60, 80, 100 µm) between the rolls. The speed of rolls was fixed at 100 rpm. The quality of the obtained samples was compared through the measurement of their electrical conductivity and mechanical strength (Supplementary Materials Table S1). In this manner, the optimal processing parameters of TRM (5-μm roll gap and a 3-step trial) were chosen for further experiments.

At the next stage, composite samples loaded with 10, 15 and/or 20 wt.% of carbon filler (graphene, CNTs, or hybrids with different G/CNT mass ratios) were fabricated by initial hand mixing and subsequent calendering with TRM processing parameters chosen at the previous step. The obtained mixtures were poured into molds and cured in an oven at 100 °C for 35 min.

**Figure 1:**
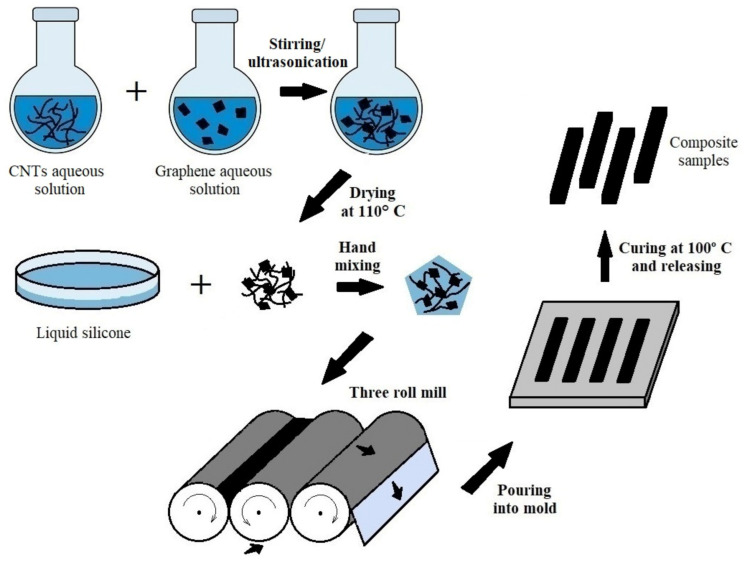
Schematic illustration of composite manufacturing process.

For illustration, the complete fabrication scheme is presented in Figure 1.

### 2.3. Characterization

The microstructure and morphology of G/CNT hybrid fillers and their silicone composites were observed using scanning electron microscopy (SEM) (Carl Zeiss: Merlin, Germany). For statistical purposes, 5 different areas were analyzed on each composite sample.

The mechanical properties of G/CNT/PDMS composites were studied by using a computerized universal testing machine (AG-50kNXD, Shimadzu Co., Kyoto, Japan). The test samples (5 specimens of each composite type) were prepared according to the ASTM D638-Standard Test Method for Tensile Properties of Plastics. The relative measurement error did not exceed 5%.

A Keithley 2400 digital multimeter (Keithley Instruments Ltd., Reading, UK) was used for high-precision resistance measurements by the four-point probe method. The dynamic electromechanical properties of the conductive composites were obtained by the electromechanical coupling test system, including a self-made stretching device and Keithley 2400 digital multimeter controlled by LabVIEW. In the electromechanical test, the gauge length was 16 mm with a loading speed of 40 mm/min. All electrical measurements were performed for 5 samples of each type, and the relative measurement error did not exceed 3%.

In order to evaluate the biocompatibility of CNT/G/PDMS composites, a human neuroblastoma SH-SY5Y cell culture was used. Cells were cultured on 10.5 cm plates (SPL Life Sciences Co., Ltd., Seoul, Korea) in EMEM with the addition of 1% penicillin-streptomycin solution, 10% FBS, and 1 µM retinoic acid to induce neuronal differentiation. The cell culture was kept in an incubator (SHELLAB, Cornelius, OR, USA) at 37 °C, 90% relative humidity, and 5% CO_2_ for 10 days. Further, the cells with the culture medium were distributed over 24-well plates (SPL Life Sciences Co., Ltd., Seoul, Korea) with or without CNT/G/PDMS samples. After 4 days, the viability evaluation was performed through the MTT test. A 0.5 mg/mL solution of the MTT reagent in a cultural medium was added to the cells. After a 2 h incubation of cells with the MTT reagent, the medium was removed from the wells and replaced with 500 µl of DMSO. Afterwards, the plates were placed into a Synergy H1 Microplate Reader (BioTek instruments, Winooski, VT, USA). The absorbance of samples was measured at the wavelengths λ = 570 nm and λ = 660 nm. Then, the absorbance value at 660 nm was subtracted from the absorbance value at 570 nm. The data were presented as percentages of the absorbance values in the control wells, which were taken as 100%. Data analysis was performed using GraphPad prism 7 software. Difference between groups was evaluated using Shapiro–Wilk normality test and *t*-test. Images of the culture were taken using a Nikon TS-100 (Nikon, Tokyo, Japan) microscope.

## 3. Results and Discussion

**Figure 2:**
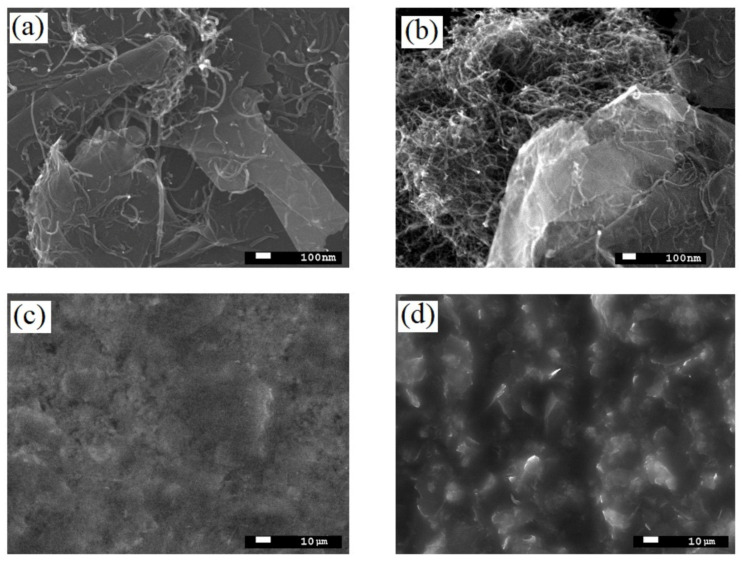
SEM images of (**a**) G/CNT (8:2, *w/w*) hybrid fillers; (**b**) example of agglomerated hybrid fillers (G/CNT (2:8, *w/w*)); (**c**) surface of 15 wt.% silicone composite with G/CNT (8:2, *w/w*) filler; (**d**) example of composite surfaces with agglomerated hybrid fillers (G/CNT (2:8, *w/w*)).

SEM analysis of G/CNT hybrid materials with various mass ratios (9:1, 8:2, 6:4, 4:6, and 2:8) demonstrated that the most uniform dispersion and homogeneity of carbon allotropes were achieved for the G/CNT mass ratio of 8:2. As can be seen from micrographs (Figure 2a), carbon nanotubes in these samples are evenly distributed on and between the graphene sheets, which prevents their restacking and agglomeration. On the contrary, SEM pictures of the hybrid samples with other G/CNT mass ratios demonstrate the presence of significant amounts of CNT and FLG agglomerates (Figure 2b). At the same time, the SEM pictures of silicone composites with G/CNT (8:2, *w/w*) fillers indicate a high uniformity of composite surfaces (Figure 2c), which is due to the even distribution of G/CNT fillers in the silicone matrix. Moreover, a significant roughness of composite surfaces caused by a high level of agglomeration is observed for samples with other G/CNT mass ratios (Figure 2d).

**Figure 3:**
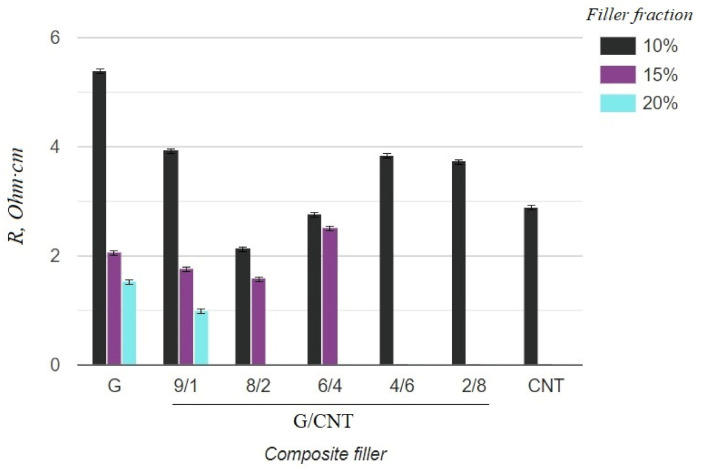
Resistivity of silicone composites with different filler fractions.

**Figure 4:**
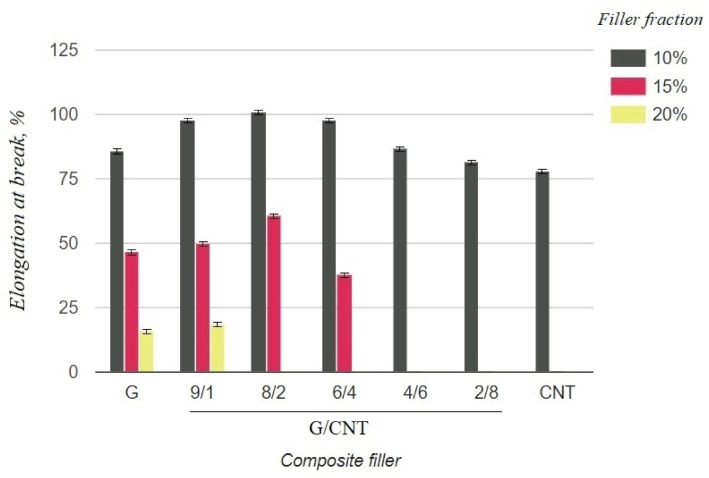
Elongation at break of silicone composites with different filler fractions.

The electrical and mechanical properties of the composite samples with various achievable concentrations (10, 15, 20 wt.%) and G/CNT ratios were investigated. The obtained results are presented in Table 1. The tabular data were also used to build diagrams illustrating the dependences of mechanical and electrical properties of silicon composites on the filler concentration and the ratio of G/CNT in hybrid fillers (Figure 3 and Figure 4).

As can be seen from the graph in Figure 3, the electrical resistivity of composites with the CNT filler has lower values than that of composites with the graphene filler at 10 wt.% concentrations, which is due to the ability of CNTs to form a better conductive network in polymer matrices. Thus, it was expected that the electrical conductivity of the hybrid G/CNT silicone composites would decrease as the ratio of graphene increased; however, it was found that the conductivity of some hybrid composites is comparable or even exceeds the conductivity of CNT/PDMS composites. This phenomenon (synergistic effect) is due to the enhancement of hybrid filler dispersibility and formation of a 1D-2D interconnection network among CNTs and graphene platelets. According to our experimental data, the synergistic effect is more dominant than the intrinsic properties of carbon fillers at the G/CNT ratios of 8:2 and 6:4, which is consistent with the results obtained for non-silicone composites [27,28]. The presence of a synergistic effect is also confirmed by the mechanical test results (Figure 4). According to these data, the elongation at break values of composites with 10 wt.% of G/CNT (8:2) and (6:4) fillers exceeds that of composites with either CNTs or graphene fillers alone. Moreover, with an increase in concentrations up to 15 wt.%, composites with G/CNT (8:2) fillers outperform composites with G/CNT (6:4) fillers in their electrical and mechanical properties, which is due to the higher bulk density of the G/CNT (8:2) filler and, consequently, its better dispersion in the silicone matrix. Thus, the silicone composites with 15 wt.% of G/CNT (8:2) hybrid filler were found to be optimal for the production of stretchable electrodes.

**Figure 5:**
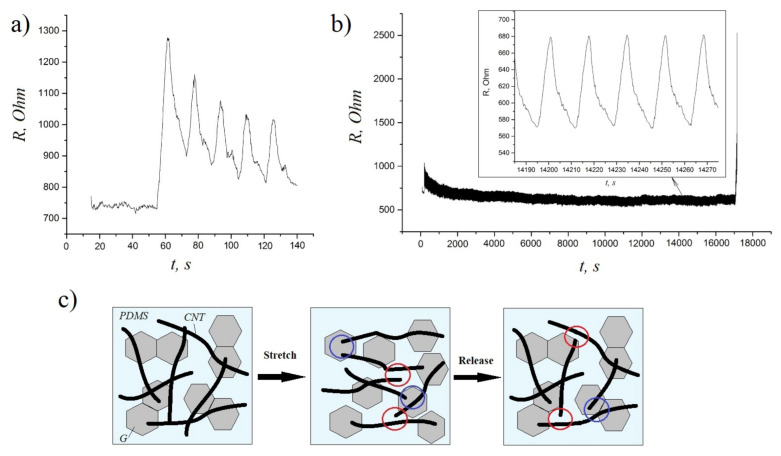
**(a**) The resistance response of hybrid composites at the initial cycles of stretching–releasing (0% to 30% strain); (**b**) the resistance response of hybrid composites during >1000 cycles of stretching–releasing (0% to 30% strain); (**c**) the schematic illustration of the mechanism underlying the conductivity of hybrid composites during stretching–releasing cycles.

To evaluate the durability and electrical stability of these composites under cyclic loadings, we also performed a series of stretching/releasing tests at 30% strain. According to the obtained results, the resistance of composite samples increased slightly during the initial cycles (Figure 5a) and then remained very stable up to 1000 cycles (see the magnifying inset of Figure 5b), which is due to the formation of a better conductive network after a period of structure modulation [29]. The schematic illustration of the mechanism underlying the conductivity of hybrid composites during stretching–releasing cycles is presented in Figure 5c. The stretching of CNT/G/PDMS composites causes some permanent damage to the CNTs networks (the red circles), which results in the conductivity loss. However, graphene sheets serve as bridges (the blue circles) between CNTs during the stretching/releasing process, so the conductive network is more easily reconstructed compared to single filler composites (CNT/PDMS and G/PDMS) [21]. Thus, the obtained results demonstrate a great potential of CNT/G/PDMS composites for flexible electronics applications.

**Figure 6:**
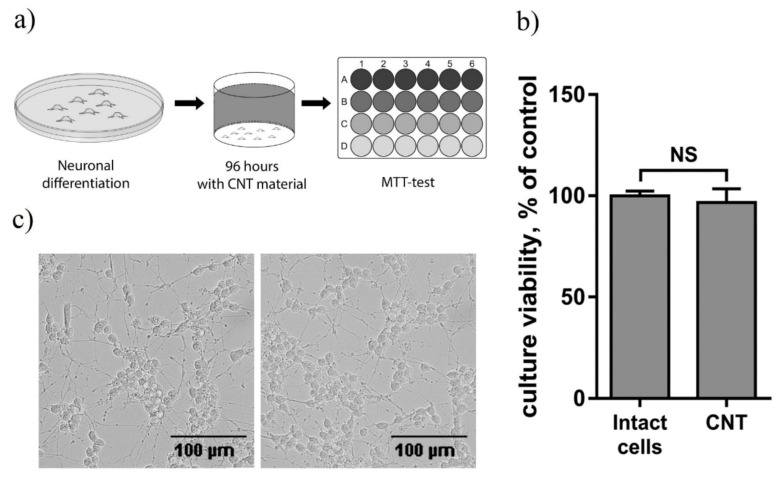
Evaluation of the CNT/G/PDMS biocompatibility on the differentiated human neuroblastoma SH-SY5Y cell line: (**a**) the experimental design; (**b**) comparison of the culture viability of the control cells and those incubated with CNT/G/PDMS cells, *n* = 8; (**c**) microphotographs of the control cells and those incubated with CNT/G/PDMS cells.

In order to evaluate the biocompatibility of CNT/G/PDMS composites, we tested their toxicity over the course of 4 days for human neuroblastoma SH-SY5Y cell culture, differentiated with 1 µM retinoic acid (Figure 6a). Then, the viability of the culture was evaluated using the MTT test and microscopy. According to the results of the MTT test, incubation of the culture for 4 days with CNT/G/PDMS samples had no significant effect on culture viability compared to the control (*p* = 0.56) (Figure 6b). Visual analysis of microphotographs of the culture revealed no differences between control cells and those incubated with CNT/G/PDMS samples (Figure 6c). Thus, we can conclude that CNT/G/PDMS composites have no toxic effect on the human cell culture and can be used for biosensors and bioelectronics applications.

## 4. Conclusions

The present work aimed to produce biocompatible G/CNT/PDMS hybrid composites characterized by high stretchability and electrical conductivity. To achieve this aim, we developed a cost-effective, easy handling, non-toxic, and scalable technique based on aqueous solution blending and calendering processes. A key feature of the proposed approach is the use of a synergistic effect of non-covalent π-π stacking interactions in the G/CNT hybrid system. Our experimental studies of G/CNT/PDMS composites with various component ratios demonstrated that the synergistic effect of CNT and graphene interactions significantly improves their dispersion in the silicone matrix and contributes to the superior electrical and mechanical properties of hybrid composites compared to composites with either of these fillers alone. Moreover, the strongest synergistic effect was observed in silicone composites with the G/CNT ratio of 8:2. These composites at a filler concentration of 15 wt.% exhibited a low level of resistivity (1.61 Ohm·cm), a high level of elasticity (elongation at break, 60%), good durability (>1000 strain cycles), and prominent electrical stability under cyclic loading at 30% strain. The cytotoxicity studies also confirmed the good biocompatibility of G/CNT hybrid silicone composites, which demonstrates their vast potential for applications as stretchable conductors in wearable electronics and biomedical electronic devices.

## Figures and Tables

**Table 1:** Properties of silicone composites with different content of carbon fillers.

Filler Type	Filler Content (wt.%)	Resistance, R (Ohm·cm)	Elongation at Break, Z (%)
Graphene (100%)	10	5.39	85.84
	15	2.07	46.68
	20	1.54	15.56
Graphene (90%) + CNT (10%)	10	3.96	97.79
	15	1.77	50.46
	20	1.01	18.88
Graphene (80%) + CNT (20%)	10	2.16	100.80
	15	1.61	60.58
Graphene (60%) + CNT (40%)	10	2.78	97.59
	15	2.53	38.36
Graphene (40%) + CNT (60%)	10	3.85	86.72
Graphene (20%) + CNT (80%)	10	3.74	81.83
CNT (100%)	10	2.90	78.38
Pristine silicone (100%)	-	2.9 × 10^14^	87.78

## Data Availability

The data presented in this study are available on request from the corresponding author.

## Supplementary Materials

The following are available online at https://www.mdpi.com/article/10.3390/nano11051143/s1, Table S1: TRM trials of PDMS composites with 10 wt.% of graphene/CNT (8:2) fillers.
