# Supplementary material for: Biocompatible, Electroconductive, and Highly Stretchable Hybrid Silicone Composites Based on Few-Layer Graphene and CNTs
Source: Nanomaterials (Basel). 2021 Apr 28;11(5):1143. doi: 10.3390/nano11051143 (PMC8145146; doi:10.3390/nano11051143)
Supplement: Supplementary file 1 [file nanomaterials-11-01143-s001.zip › nanomaterials-1179333-supplementary.pdf]

Supplementary Materials

Analysis of TRM Processing Parameters

The results of experimental studies on the influence of three roll mill (TRM) processing parameters upon electrical and mechanical properties of hybrid silicone composites are presented in Table 1.

Table S1. TRM trials of PDMS composites with 10 wt.% of graphene/CNT (8:2) fillers

Processing Trial	Processing parameters		Resistance (Ohm cm)	Elongation at break (%)
	Number of steps	Gap (μm)		
A	3	5	2.16	100.80
B	6	5	4.00	95.61
C	3	10	2.96	94.86
D	3	20	4.53	92.23
E	3	40	5.50	90.83
F	3	60	7.08	90.34
G	3	80	9.40	88.80
H	3	100	10.14	85.08
I	6	100	14.81	83.34

According to the obtained results, the best electrical conductivity and mechanical strength were achieved using the TRM processing parameters of trial “A”. As can be seen from the provided data, electrical conductivity and mechanical strength improve with a decrease in the gap between the mill rolls. The microscopic examination of the samples also confirmed that a rather good distribution of filler in the silicone matrix can be achieved at the minimal roll gaps (5, 10 μm). However, the addition of further steps (trials B and I) leads to deterioration of electrical and mechanical properties, which is most likely due to the crushing of graphene platelets and nanotubes into smaller fragments. In this case, the damage of the filler overbalances the improvement of filler dispersion. Thus, in order to achieve homogeneous filler distribution as well as to reduce the risk of filler damage, a 5-μm roll gap and a 3-step trial were chosen as TRM processing parameters for further experiments.
